# Ferulic Acid and P-Coumaric Acid Synergistically Attenuate Non-Alcoholic Fatty Liver Disease through HDAC1/PPARG-Mediated Free Fatty Acid Uptake

**DOI:** 10.3390/ijms232315297

**Published:** 2022-12-04

**Authors:** Kaili Cui, Lichao Zhang, Xiaoqin La, Haili Wu, Ruipeng Yang, Hanqing Li, Zhuoyu Li

**Affiliations:** 1 Institute of Biotechnology, The Key Laboratory of Chemical Biology and Molecular Engineering of Ministry of Education, Shanxi University, Taiyuan 030006, China; 2 Institutes of Biomedical Sciences, Shanxi University, Taiyuan 030006, China; 3 College of Life Science, Shanxi University, Taiyuan 030006, China

**Keywords:** ferulic acid, p-coumaric acid, NAFLD, free fatty acid uptake

## Abstract

Non-alcoholic fatty liver disease (NAFLD) is the most common liver disease and has become a growing public health concern worldwide. Polyphenols may improve high-fat diet (HFD)-related NAFLD. Our previous study found that ferulic acid (FA) and p-coumaric acid (p-CA) were the polyphenols with the highest content in foxtail millet. In this study, we investigated the mechanism underlying the impact of ferulic acid and p-coumaric acid (FA/p-CA) on non-alcoholic fatty liver (NAFLD). The association of FA and p-CA with fatty liver was first analyzed by network pharmacology. Synergistic ameliorating of NAFLD by FA and p-CA was verified in oleic acid (OA) and palmitic acid (PA) (FFA)-treated hepatocytes. Meanwhile, FA/p-CA suppressed final body weight and TG content and improved liver dysfunction in HFD-induced NAFLD mice. Mechanistically, our data indicated that FA and p-CA bind to histone deacetylase 1 (HDAC1) to inhibit its expression. The results showed that peroxisome proliferator activated receptor gamma (PPARG), which is positively related to HDAC1, was inhibited by FA/p-CA, and further suppressed fatty acid binding protein (FABP) and fatty acid translocase (CD36). It suggests that FA/p-CA ameliorate NAFLD by inhibiting free fatty acid uptake via the HDAC1/PPARG axis, which may provide potential dietary supplements and drugs for prevention of NAFLD.

## 1. Introduction

Non-alcoholic fatty liver disease (NAFLD) has become one of the serious diseases endangering global health [1,2]. NAFLD, which is the hepatic manifestation of metabolic syndrome, encompasses a spectrum of liver pathologies ranging from steatosis to non-alcoholic steatohepatitis (NASH), characterized by hepatocyte injury and fibrosis, and, eventually, progression to cirrhosis and hepatocellular carcinoma [3,4]. The progression of NAFLD will lead to irreversible liver damage and a high fatality rate [5]. Therefore, intervention in non-alcoholic fatty liver disease possesses very important significance. Currently, treatment for NAFLD is mainly to lose weight through bariatric surgery or non-pharmacological management, such as a healthy lifestyle/diet [6]. There are fewer effective approved drugs for treatment of NAFLD [7].

Polyphenols are a large group of naturally occurring compounds found in a wide range of plant-derived cereals, fruits, and vegetables. Accumulating evidence suggests that polyphenols possess favorable effects against NAFLD through a variety of molecular mechanisms, including inhibition of free fatty acid uptake and lipogenesis, activation of β- oxidation, as well as inhibiting adipocyte differentiation [8]. Foxtail millet is a traditional nutritional food in China. It has been reported that polyphenol nutrients from foxtail millet inhibit LDL cholesterol and thereby reduce lipid content in diabetic rats [9]. Our previous study found that FA and p-CA were the two most abundant bound polyphenols from foxtail millet [10]. FA, which has been approved for therapies targeting cardiovascular problems, has wide anti-oxidant [11], anti-inflammatory [12], and hepatoprotective therapeutic effects [13]. Studies show that p-CA has a broad range of biological activities, such as anti-inflammatory, antioxidant, antifibrotic, and antiadipogenic activities [14]. However, the protective effects of FA/p-CA against NAFLD and the underlying mechanisms remain unclear. 

With the development of systems biology, network pharmacology emerges [15], which establishes effective targeted, multi-component therapeutic networks and has become an appropriate method to study biologically active nutrients and their mechanisms [16]. In this study, the ameliorating effects of FA/p-CA on fatty liver were evaluated by network pharmacology analysis and in vitro and in vivo experiments. Our results may provide potential dietary supplements and drugs for NAFLD treatment.

## 2. Results

### 2.1. Prediction of the Association of FA/p-CA with Non-alcoholic Fatty Liver Disease

**Figure 1:**
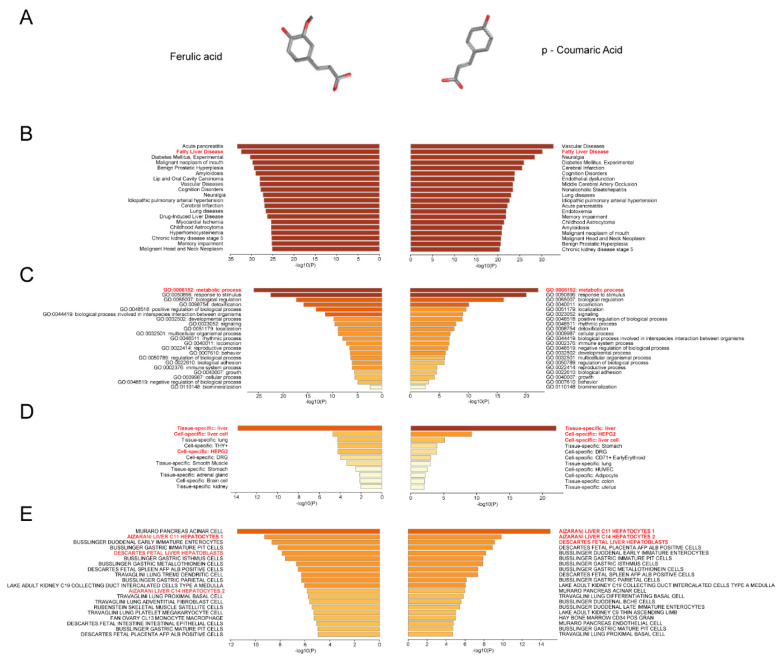
Prediction of the association of FA/p-CA with non-alcoholic fatty liver disease. (**A**) Chemical structure of FA and p-CA. FA and p-CA target genes were imported into the database. (**B**–**E**) Summary of enrichment analysis in DisGeNET (**B**). The top-level gene ontology biological processes (**C**). Summary of enrichment analysis in PaGenBase (**D**) and Cell Type Signatures (**E**). Colored by *p*-values, the color becomes lighter as the *p*-value increases.

In this part, the relationship between FA and p-CA targets with fatty liver was first analyzed using network pharmacology. Based on the structures of FA and p-CA (Figure 1A), their target genes were predicted using the SEA database and DRAR-CPI server, and 183 FA target genes and 153 p-CA target genes were obtained. Then, these target proteins of FA or p-CA were imported into the Metascape database (www.metascape.org (accessed on 21 October 2022)) for gene enrichment analysis. The results showed that the target genes of FA and p-CA were enriched in fatty liver disease (Figure 1B), involved in metabolic pathways (Figure 1C). According to PaGenBase and Cell Type Signatures analysis, both of them were most enriched in liver tissue and hepatocytes (Figure 1D,E). 

### 2.2. FA/p-CA Treatment Suppresses FFA-Induced Lipid Accumulation In Vitro

**Figure 2:**
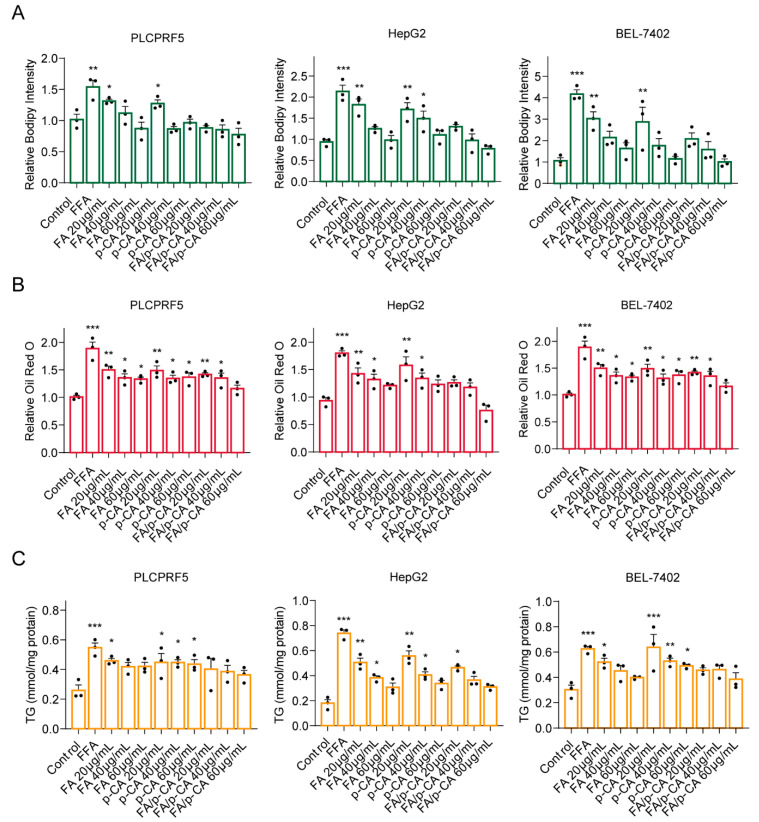
FA/p-CA treatment suppresses FFA-induced lipid accumulation in vitro. (**A**,**B**) After stimulation with or without FFA, PLCPRF5, HepG2, and BEL-7402 cells were exposed to FA, p-CA, and FA/p-CA, respectively, and then stained with BODIPY (**A**) and Oil Red O (**B**) to detect cellular lipid accumulation. The content ratio of p-CA and FA in the FA/p-CA was 1:1.13. (**C**) TG content in PLCPRF5, HepG2, and BEL-7402 cells of each group treated with FA, p-CA, and FA/p-CA. The data are presented as the mean ± SD. Compared to control, * *p* < 0.05, ** *p* < 0.01, *** *p* < 0.001.

Based on the consequences of network pharmacology, we speculate that FA/p-CA may have a synergistic effect in regulating NAFLD. To prove the hypothesis, the inhibitory effects of FA, p-CA, and FA/p-CA on hepatocytes stimulated with FFA were examined. Bodipy and Oil Red O staining showed that cellular lipid accumulation was significantly decreased by FA, p-CA, and FA/p-CA (Figure 2A,B). The determination of TG content further verified its inhibitory effect on lipid accumulation (Figure 2C). Interestingly, the inhibitory effect of FA/p-CA was far more than that of the single component. All these findings demonstrate that FA/p-CA have a synergistic effect in improving NAFLD. 

### 2.3. FA/p-CA Attenuate HFD-Induced Hepatic Injury and Steatosis In Vivo

**Figure 3:**
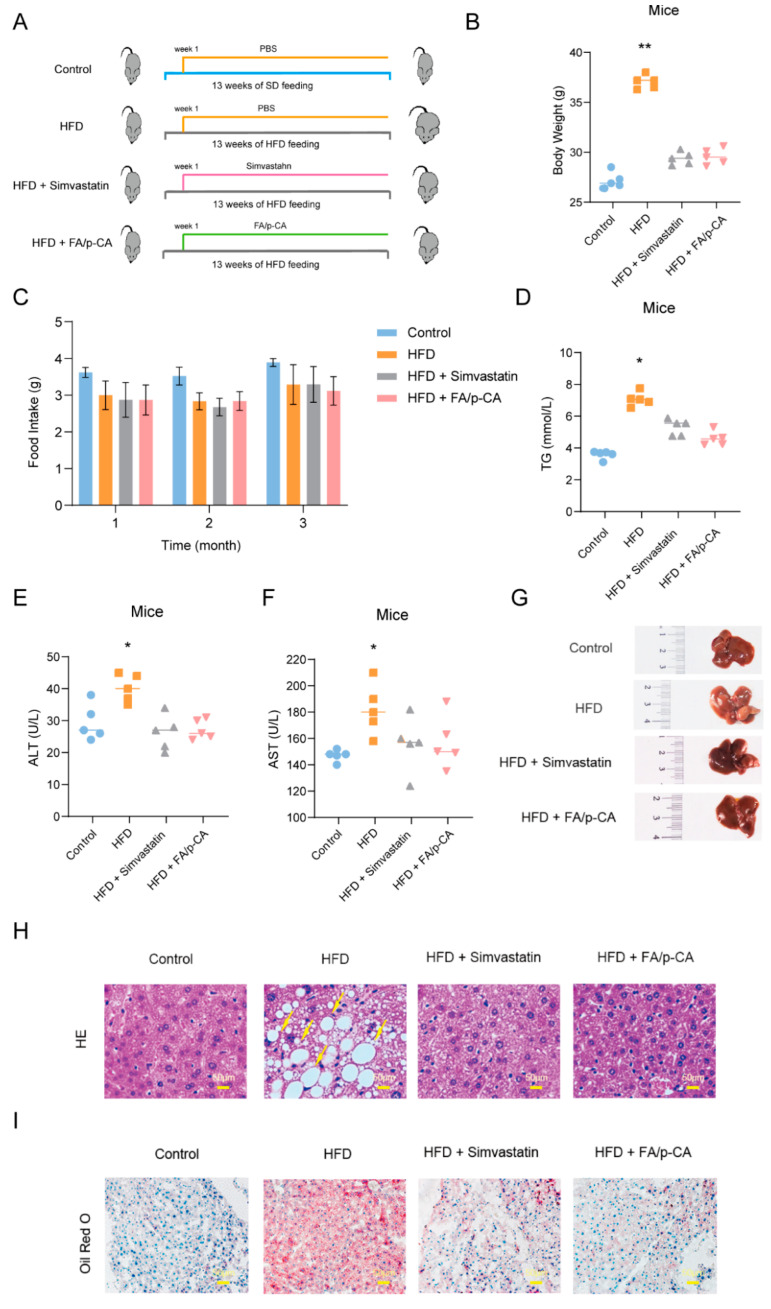
FA/p-CA attenuate HFD-induced hepatic injury and steatosis in vivo. (**A**) An overview of the experimental design of HFD-induced NAFLD mice model. FA/p-CA was administered by gavage and the content ratio of p-CA and FA in the FA/p-CA was 1:1.13. (**B**) Body weight. (**C**) Food intake. (**D**) Serum total TG. (**E**) Serum ALT. (**F**) Serum AST. (**G**) Macroscopic structure of the liver. (**H**,**I**) Representative images of H&E (**H**) and Oil Red O (**I**) staining at 40 × magnification. Yellow arrows are used to mark steatosis and hepatocyte ballooning. Compared to control, * *p* < 0.05, ** *p* < 0.01.

We then evaluated the ameliorating effect of FA/p-CA on fatty liver in an HFD-induced NAFLD mice model. According to the procedure shown in Figure 3A, we constructed the HFD-induced NAFLD mice model and evaluated the improvement effect of FA/p-CA on fatty liver in vivo. The results showed that, after 13 weeks of HFD feeding, the HFD-diet-fed mice showed a significant increase in body weight, which was prevented by administration of FA/p-CA and simvastatin (Figure 3B). Food intake was not altered by FA/p-CA and simvastatin (Figure 3C). Meanwhile, FA/p-CA and simvastatin supplement reduced the levels of serum total TG, as well as the levels of serum ALT and AST (Figure 3D–F). Furthermore, both morphologic and histological examinations of liver exhibited striking alteration in liver color, more steatosis, and hepatocyte ballooning in the HFD mice. Moreover, FA/p-CA and simvastatin treatment alleviated HFD-induced lipid accumulation (Figure 3G,H). Oil Red O staining of histological examination further confirmed that FA/p-CA and simvastatin improve HFD-induced lipid accumulation (Figure 3I). Taken together, the data demonstrate that FA/p-CA protect against HFD-induced hepatic steatosis.

### 2.4. The Potential Pathways of FA p-CA Targets

**Figure 4:**
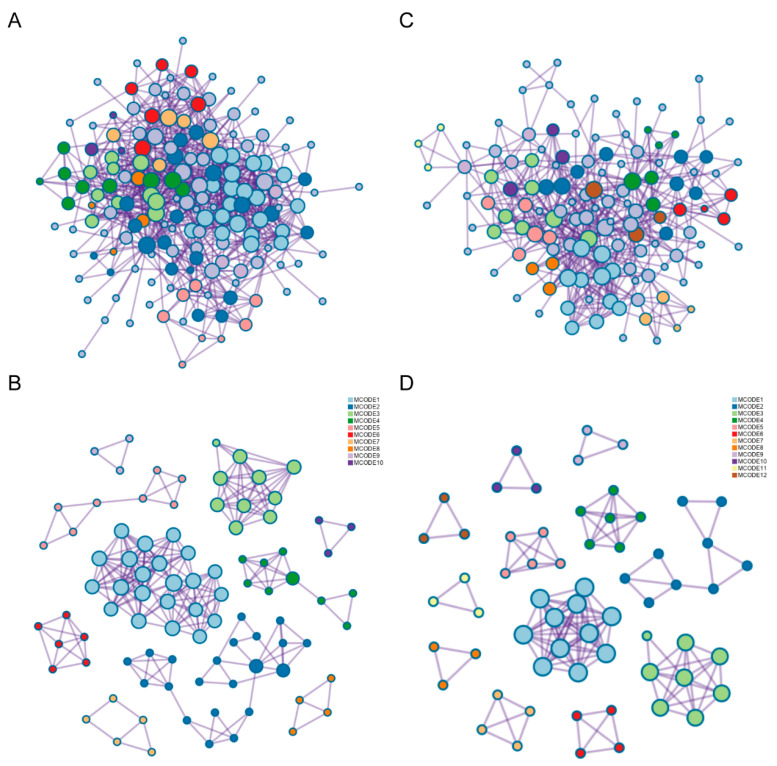
The potential pathways of FA/p-CA targets. (**A**,**B**) Protein–protein interaction network and MCODE components identified in FA targets. (**C**,**D**) Protein–protein interaction network and MCODE components identified in p-CA targets.

To further explore the molecular mechanism of FA/p-CA synergistic improvement on NAFLD, the target proteins of FA and p-CA were used to construct protein–protein interaction (PPI) networks and clustered using its built-in MCODE algorithm from Metascape database (Figure 4A,B). According to the value of Log10(P), we found that FA target genes were enriched in the PID retinoic acid pathway and nuclear receptor transcription pathway (Table 1). Further, p-CA target genes were enriched in NOTCH1 intracellular domain regulate transcription (Table 2). 

### 2.5. HDAC1 Is Involved in Amelioration of NAFLD by FA/p-CA

**Figure 5:**
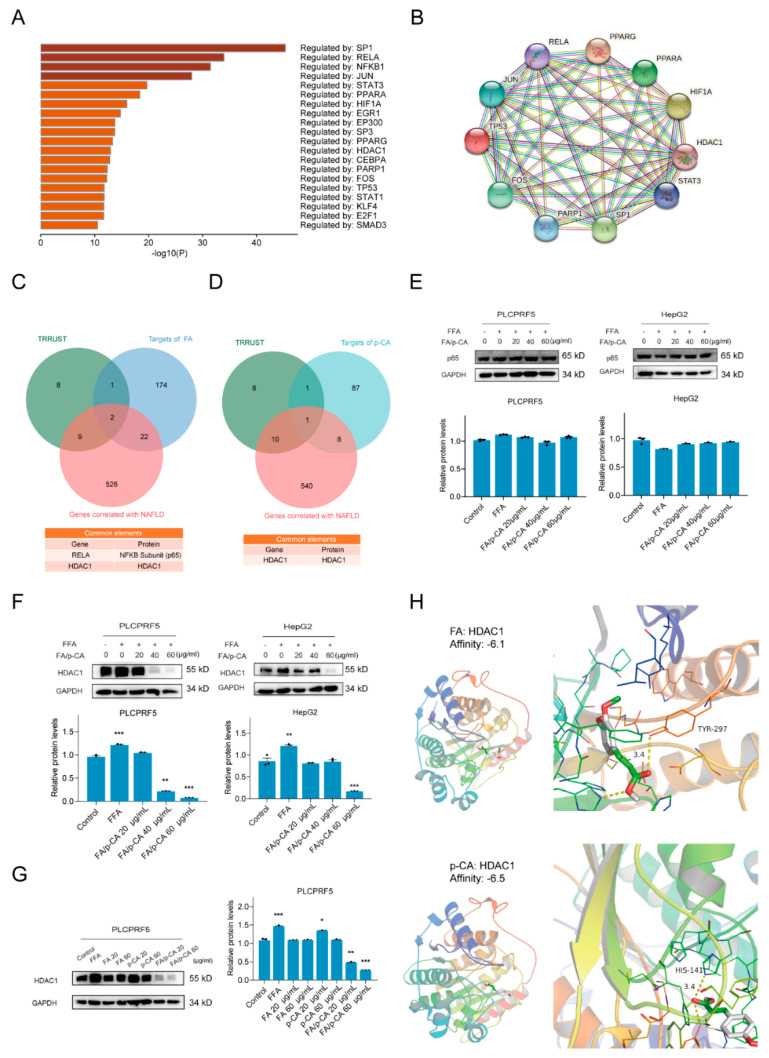
HDAC1 is involved in the amelioration of NAFLD by FA/p-CA. (**A**) Summary of enrichment for TRRUST categories when 558 NAFLD-related targets were uploaded to Metascape, colored with *p*-values; the color becomes lighter as the *p*-value increases. (**B**) PPI network of transcriptional regulators. (**C**,**D**) FA (**C**) or p-CA (**D**) target genes were intersected with TRRUST and NAFLD-related genes by the VENNY tool. (**E**,**F**) The expressions of p65 (**E**) and HDAC1 (**F**) in FFA-stimulated PLCPRF5 and HepG2 cells were detected by different concentrations of FA/p-CA (0, 20, 40, 60 μg/mL). Relative protein levels were analyzed using ImageJ software. Data represented as mean ± SD. (**G**) The PLCPRF5 cells were, respectively, exposed to increasing concentrations of FA, p-CA, and FA/p-CA, and then the expression of HDAC1 was determined by Western blot. Expression of GAPDH was used as the internal control. A representative result from three independent experiments was shown. Relative protein levels of HDAC1 were analyzed using ImageJ software. Data represented as mean ± SD. Compared to control, * *p* < 0.05, ** *p* < 0.01, *** *p* < 0.001. (**H**) Molecular docking model of FA or p-CA and HDAC1.

According to detection of the PPI network of FA and p-CA target genes, we hypothesized that FA/p-CA regulate gene expression at the transcriptional level to improve NAFLD. To further demonstrate this finding, 558 NAFLD-related targets were retrieved through the GeneCards database and HOME-NCBI-GENE database. Then, we uploaded these genes to Metascape to screen for their transcriptional regulators through the TRRUST category (Figure 5A). Transcriptional regulators were imported into the STRING database (https://cn.string-db.org (accessed on 21 October 2022)) to construct a PPI network (Figure 5B). Further, FA or p-CA target genes were intersected with TRRUST and NAFLD-related genes, respectively, to determine NAFLD transcription-associated proteins targeted by FA or p-CA (Figure 5C,D). We next explored expression of these proteins in the presence of FA/p-CA. The results showed no significant change in p65 (Figure 5E), while HDAC1 was significantly inhibited (Figure 5F). Consistent with its effect on NAFLD, FA/p-CA inhibited HDAC1 expression better than the single component (Figure 5G). The interaction of FA and p-CA with HDAC1 protein was predicted by molecular docking experiments. The results showed that FA formed hydrogen bonds with the H141 site of HDAC1, while p-CA had hydrogen bonds with Y297 (Figure 5H). The above data indicated that FA and p-CA bind to different sites of HDAC1 to inhibit its expression and coordinately improve NAFLD.

### 2.6. FA/p-CA Inhibit Hepatic Lipid Uptake Via the HDAC1/PPARG Axis

**Figure 6:**
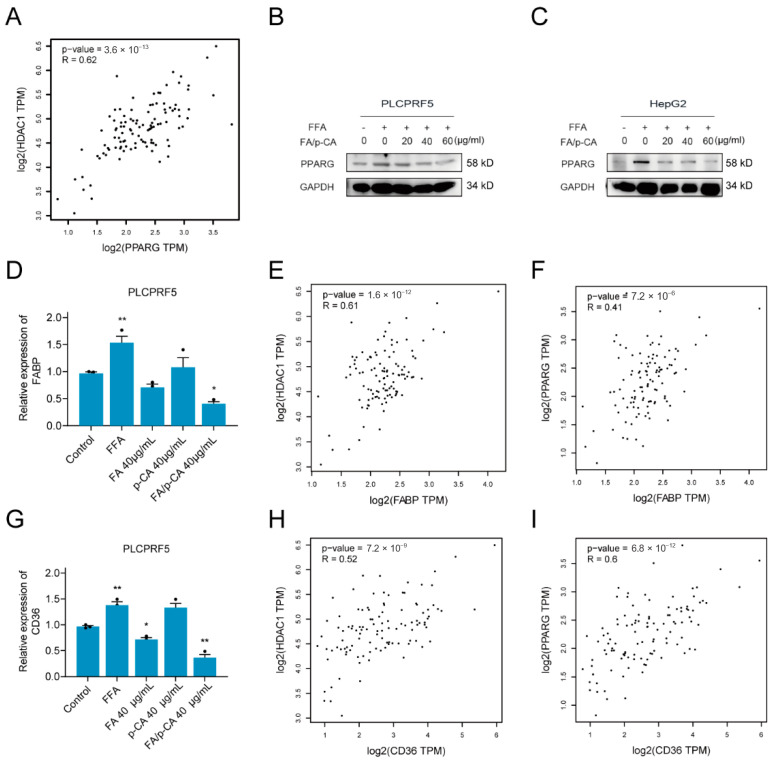
FA/p-CA inhibit hepatic lipid uptake via the HDAC1/PPARG axis. (**A**) The correlation analysis between HDAC1 and PPARG. (**B**,**C**) The PLCPRF5 (**B**) and HepG2 (**C**) cells were, respectively, exposed to increasing concentrations of FA, p-CA, and FA/p-CA, and then the expression of PPARG was determined by Western blot. (**D**) The PLCPRF5 cells were, respectively, exposed to FA, p-CA, and FA/p-CA, and then the expression of FABP was determined by qRT-PCR. Data represented as mean ± SD. (**E**,**F**) The correlation analysis between FABP and HDAC1 (**E**) or PPARG (**F**). (**G**) The PLCPRF5 cells were, respectively, exposed to FA, p-CA, and FA/p-CA, and then the expression of CD36 was determined by qRT-PCR. Data represented as mean ± SD. Compared to control, * *p* < 0.05, ** *p* < 0.01. (**H**,**I**) The correlation analysis between CD36 and HDAC1 (N) or PPARG (O).

**Figure 7:**
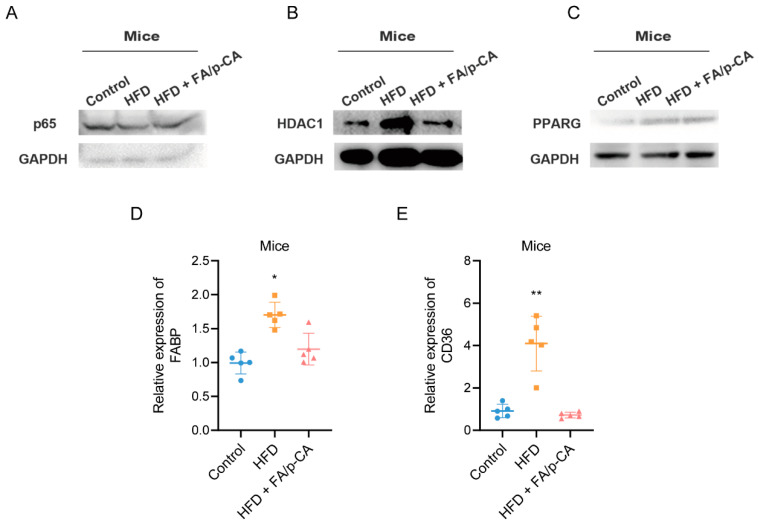
FA/p-CA inhibit hepatic lipid uptake via the HDAC1/PPARG axis in vivo. (**A**–**C**) Mice were treated with FA/p-CA, then expressions of p65 (**A**), HDAC1 (**B**), and PPARG (**C**) were determined by Western blot. (**D**,**E**) FA/p-CA were administered by gavage; expressions of FABP and CD36 were determined by qRT-PCR. Data represented as mean ± SD. Compared to control, * *p* < 0.05, ** *p* < 0.01.

Fatty acid metabolism disorders are closely related to non-alcoholic fatty liver disease, and intervention of fatty acid metabolism is an effective way to improve NAFLD. Based on this, GEPIA2 was applied to analyze the correlation of HDAC1 with key transcription factors of fatty acid metabolism, including peroxisome proliferator activated receptor alpha (PPARA), PPAR, sterol regulatory element binding transcription factor 1 (SREBF1), sterol regulatory element binding transcription factor 2 (SREBF2), nuclear receptor subfamily 1 group H member 3 (NR1H3), CCAAT enhancer binding protein alpha (CEBPA), and forkhead box A2 (FOXA2). The results showed that HDAC1 was positively correlated with PPARG (Figure 6A) but not with other transcription factors (Figure S1A–F). Consistent with HDAC1 protein, the expression of PPARG decreased after FA/p-CA intervention (Figure 6B,C). PPARG is a transcription factor that regulates free fatty acid uptake, which is regulated by CD36 and FABP. The expressions of these mRNA were further detected after FA, p-CA, and FA/p-CA treatment. The results showed that the expression of FABP and CD36 was decreased, and the effect of FA/p-CA was more significant compared to the single component (Figure 6D,G). A correlation analysis of lipid uptake genes with HDAC1 and PPARG was performed. The results showed that FABP (Figure 6E,F) and CD36 (Figure 6H,I) had a positive correlation with HDAC1/PPARG. Furthermore, we verified the effect of FA/p-CA on the HDAC1/PPARG signaling pathway in vivo. The results showed that, after FA/p-CA supplement, the expression of p65 protein was not significantly changed (Figure 7A), and the expression of HDAC1 (Figure 7B) and PPARG (Figure 7C) protein was inhibited. Moreover, FA/p-CA treatment strikingly repressed the elevation of HFD-induced FABP (Figure 7D) and CD36 (Figure 7E) mRNA levels. These results are consistent with the results of cellular experiments. The above results suggest that FA/p-CA inhibit hepatic free fatty acid uptake via the HDAC1/PPARG axis.

## 3. Discussion

Lifestyle changes are beneficial in the treatment of NAFLD [17]. However, poor adherence to lifestyle changes makes NAFLD management a daunting task. Pharmaceutical therapy is an important part of treating NAFLD, but no drug has been specifically approved to date [18]. FA is a drug approved for treatment of atherosclerosis and has the effect of preventing lipid peroxidative damage [19,20]. In this study, FA was found to ameliorate NAFLD by inhibiting lipid accumulation through network pharmacology analysis and in vitro experiments. Cereal polyphenols have a variety of biological activities, such as antioxidant, anti-inflammatory, and antifibrotic [21,22]. FA and p-CA are two kinds of polyphenols that account for a higher proportion of foxtail millet polyphenols. Interestingly, the study found that p-CA enhances the improvement effect of FA on NAFLD. FA and p-CA demonstrate a synergistic effect on improvement in fatty liver.

A series of animal models have been established to study NAFLD [23]. In particular, induction of NAFLD by HFD in C57BL/6 mice is the most widely used rodent model [24]. This diet rapidly induced NAFLD in rodents, and male C57BL/6 mice had histological features most similar to those observed in human NAFLD compared to other models [25]. In our study, HFD-fed mice exhibited classic features of NAFLD, such as steatosis and hepatocyte ballooning. Furthermore, FA/p-CA were able to improve HFD-induced lipid accumulation, which was associated with reduced TG content. In general, in vivo experiments further verified that FA/p-CA improved NAFLD by inhibiting lipid accumulation. 

According to the results of the PPI network of FA and p-CA target genes, FA and p-CA target genes were enriched in transcriptional regulatory pathways. Therefore, we screened the transcriptional regulators of fatty liver. We found that HDAC1 is targeted by FA/p-CA. Further study found that the combined effect of FA and p-CA significantly inhibited expression of HDAC1. According to the results of molecular docking, FA binds to the H141 site of HDAC1, which is associated with its deacetylation activity [26,27], inhibiting its activity and promoting its degradation. Binding of p-CA to its Y297 site [26], which regulates HDAC1 ubiquitination activity, promotes its ubiquitination degradation. Taken together, FA and p-CA may bind to different sites of HDAC1 to synergistically inhibit its expression. 

Disorders of lipid metabolism were significantly associated with fatty liver [28]. Studies have shown that HDAC1 mediates development of NAFLD by promoting lipid accumulation [29,30]. Therefore, the lipid metabolism transcription factors related to HDAC1 were screened. It was found that PPARG, a transcription factor of lipid uptake, was positively correlated with HDAC1. Further study showed that, consistent with HDAC1, expression of PPARG and fatty acid uptake genes decreased after FA/p-CA intervention. Additionally, HDAC1/PPARG was correlated with CD36 or FABP. CD36 is a fatty acid translocase [31] and FABP is a fatty acid binding protein [32]; both of them are involved in the process of lipid uptake. In conclusion, FA/p-CA can inhibit lipid uptake through the HDAC1/PPARG axis and thereby improve fatty liver. 

## 4. Martials and Methods 

### 4.1. Materials

RPMI 1640 medium and fetal bovine serum (FBS) were from Biological Industries (BI, Israel); ferulic acid (FA) and p-coumaric acid (p-CA) were purchased from Victory Biological Technology Co., Ltd. (Sichuan, China). The content ratio of p-CA and FA in the FA/p-CA was 1:1.13 [10]. TG test kit was purchased from NanJing JianCheng Bioengineering Institute (Nanjing, China). BODIPY, palmitic acid, oleic acid, and Oil Red O assay kit were obtained from Solarbio (Beijing, China). Antibodies for HDAC1, PPARG, NF-κB (p65), and glyceraldehyde-3-phosphate dehydrogenase (GAPDH) were from Bioss (Beijing, China). All chows were produced by Xietong pharmaceutical bio-engineering Co., Ltd (Nanjing, China). The RNAiso Plus was purchased from Takara (Shiga, Japan). All-in-one First Strand cDNA Synthesis Kit Ⅱand 2×SYBR Green qPCR Master Mix were from Sevenbio (Beijing, China). 

### 4.2. Cell Culture and Steatosis Induction

Cell lines HepG2, PLCPRF5 and BEL-7402, which were obtained from the Chinese Type Culture Collection (Shanghai, China), were cultured in RPMI 1640 medium containing 10% FBS and placed in an incubator at 37 °C with 5% CO_2_. For Steatosis induction, HepG2 and PLCPRF5 cells were treated with 1 mM FFA for 24 h, and BEL-7402 cells were treated with 0.5 mM FFA for 24 h. 

### 4.3. Bodipy and Oil Red O Staining

Cell monolayers were fixed in 4% paraformaldehyde for 30 min and incubated with Bodipy and Oil Red O for 15 min at room temperature. Oil Red O bound to cell lipid droplets was extracted with isopropanol, and its OD value was measured at OD510. Wallac victor was used to detect the fluorescence value of BODIPY to check the intracellular lipid accumulation.

### 4.4. Triglyceride Assay

The cells were inoculated in a 6-well plate, and cells were collected after treating with FA, p-CA, and FA/p-CA for 48 h. Cell lysate was used to determine the TG content. The methods of measuring TG were according to the manufacturer’s instructions.

### 4.5. Mice Experiment

A total of 20 male C57BL/6J mice (5 weeks old), weighing 20 ± 5 g, were purchased from Beijing Vital River Laboratory Animal Technology Co., Ltd. This animal experiment was approved by the “Principles of Laboratory Animal Care” formulated by the National Institutes of Health and the Ethics Committee of Animal Experimentation of Shanxi University.

After a 1-week acclimation period, mice were randomly divided into four groups (Figure 3A), including one control group and three treatment groups (five mice per group). One control group was fed a normal fat diet, and three treatment groups were fed HFD (60% fat, 20% carbohydrate, and 20% protein) [33,34], one of which was given an equal volume of water, one was intragastrically administered simvastatin (5 mg kg^−1^ day^−1^), and one group was given intragastric FA/p-CA (30 mg kg^−1^ day^−1^). At the end of the study, all mice were anesthetized with pentobarbital sodium (80 mg/kg) and liver tissues were immediately removed, weighed, and frozen in liquid nitrogen for further analysis.

### 4.6. Histological Examinations

After the liver tissue was fixed in 10% formalin, paraffin sections were stained with hematoxylin–eosin (H&E) to evaluate the morphology of the samples, while frozen sections were stained with Oil Red O to visualize the lipid droplets.

### 4.7. Western Blot

PLCPRF5 and HepG2 cells were inoculated in 6-well plates and collected. The total protein was extracted and the protein concentration was measured by BCA assay. Then, 60 μg protein was added to each well and separated by SDS-PAGE. The above proteins were transferred to PVDF membrane. The membrane was further blocked and incubated overnight at 4 °C with HDAC1 (rabbit), PPARG (rabbit), NF-κB (p65) (mouse), and GAPDH (rabbit) antibodies. The corresponding secondary antibodies were incubated for 2 h at room temperature the following day. Finally, the expression of target proteins was observed by automatic chemiluminescence imaging instrument. Relative protein levels were analyzed using ImageJ software. Expression of GAPDH was used as the internal control.

### 4.8. Quantitative RT-PCR

The total RNA was extracted from cells with Trizol reagent. For cDNA synthesis, 500 ng RNA was reverse-transcribed using EasyScript First-Strand cDNA Synthesis SuperMix. The mRNA levels were quantified using qRT-PCR. The cDNA level for each gene was normalized to GAPDH mRNA levels. All primers used in the experiment are shown in Table 3.

### 4.9. Molecular Docking

The crystal structure of HDAC1 (ID: 4BKM) was obtained from the protein database (PDB, http://www.rcsb.org/ (accessed on 21 October 2022)) and downloaded in PDB format. The small molecule structure was downloaded from PubChem. Autodock Vina [35] was used to simulate the combination of FA (Compound CID: 445858) or p-CA (Compound CID: 637542) with HDAC1.

### 4.10. Statistical Analysis

The data were presented as the mean ± standard deviation of three independent experiments (mean ± SD); data error bar indicated standard deviation. Continuous variables (N ≥ 3 groups) were analyzed by single factor analysis of variance (ANOVA), and values of *p* < 0.05 indicated that there was significant difference, while *p* < 0.01 indicated that the difference was highly significant compared with control.

## 5. Conclusions

In this study, the improvement effect of FA/p-CA on fatty liver was verified in various aspects through network pharmacology experiments and in vitro and in vivo experiments. Our study indicates that FA/p-CA are promising drugs for potential clinical applications in the future.

## Figures and Tables

**Table 1:** MCODE components identified in FA target genes.

MCODE	GO	Description	Log10(P)
MCODE_1	R-HSA-190840	Microtubule-dependent trafficking of connexons from Golgi to the plasma membrane	−14.9
	R-HSA-190872	Transport of connexons to the plasma membrane	−14.7
	R-HSA-389977	Post-chaperonin tubulin folding pathway	−14.3
MCODE_2	GO:1901615	organic hydroxy compound metabolic process	−12.7
	hsa00350	Tyrosine metabolism	−10.4
	GO:0010817	regulation of hormone levels	−9.1
MCODE_3	M207	PID RETINOIC ACID PATHWAY	−19.3
	R-HSA-383280	Nuclear Receptor transcription pathway	−17.5
	M162	PID RXR VDR PATHWAY	−16.3
MCODE_4	WP167	Eicosanoid synthesis	−16.6
	WP5122	Prostaglandin and leukotriene metabolism in senescence	−16.2
	GO:0019369	arachidonic acid metabolic process	−14.5
MCODE_5	R-HSA-211859	Biological oxidations	−17.1
	WP702	Metapathway biotransformation Phase I and II	−14.6
	R-HSA-156580	Phase II—Conjugation of compounds	−13.3
MCODE_6	hsa05130	Pathogenic Escherichia coli infection	−13.1
	hsa05417	Lipid and atherosclerosis	−12.9
	hsa05131	Shigellosis	−12.6
MCODE_7	M186	PID PDGFRB PATHWAY	−6.1
	R-HSA-1280215	Cytokine Signaling in Immune system	−5.8
	GO:0030100	regulation of endocytosis	−5.5
MCODE_8	WP272	Blood clotting cascade	−8.8
	WP558	Complement and coagulation cascades	−7.5
	GO:0030193	regulation of blood coagulation	−7.4
MCODE_9	WP702	Metapathway biotransformation Phase I and II	−6.6

**Table 2:** MCODE components identified in p-CA target genes.

MCODE	GO	Description	Log10(P)
MCODE_1	R-HSA-2122947	NOTCH1 Intracellular Domain Regulates Transcription	−27.4
	R-HSA-2894858	Signaling by NOTCH1 HD+PEST Domain Mutants in Cancer	−26.5
	R-HSA-2894862	Constitutive Signaling by NOTCH1 HD+PEST Domain Mutants	−26.5
MCODE_2	R-HSA-1592389	Activation of Matrix Metalloproteinases	−7
	M174	PID UPA UPAR PATHWAY	−6.7
	WP534	Glycolysis and gluconeogenesis	−6.6
MCODE_3	GO:0046394	carboxylic acid biosynthetic process	−6.4
	GO:0016053	organic acid biosynthetic process	−6.4
	GO:1901607	alpha-amino acid biosynthetic process	−6.2
MCODE_4	R-HSA-418594	G alpha (i) signaling events	−11.9
	R-HSA-373076	Class A/1 (Rhodopsin-like receptors)	−11.8
	R-HSA-500792	GPCR ligand binding	−10.9
MCODE_6	R-HSA-6798695	Neutrophil degranulation	−7.2
	R-HSA-1474244	Extracellular matrix organization	−5.4
MCODE_7	hsa00982	Drug metabolism—cytochrome P450	−10.5
	hsa00980	Metabolism of xenobiotics by cytochrome P450	−10.4
	R-HSA-211859	Biological oxidations	−8.5
MCODE_8	R-HSA-383280	Nuclear receptor transcription pathway	−8.3
MCODE_9	WP702	Metapathway biotransformation Phase I and II	−6.6
MCODE_11	R-HSA-6798695	Neutrophil degranulation	−5.4

**Table 3:** Primers sequences used for qRT-PCR.

Genes	Sequences (5′-3′)	
FABP	forward primer:	reverse primer:
	TGGCGTTTGACAGCACTTGG	AGCTTCAAATTGTCATGAGCTGCA
CD36	forward primer:	reverse primer:
	TCTCAATCTGGCTGTGGCAG	CAGGGTACGGAACCAAACTCA
GAPDH	forward primer:	reverse primer:
	GCACCGTCAAGGCTGAGAAC	TGGTGAAGAACGCCAGTGGA

## Data Availability

All data needed to evaluate the conclusions in the paper are present in the paper.

## Supplementary Materials

The following supporting information can be downloaded at: https://www.mdpi.com/article/10.3390/ijms232315297/s1.

## References

[1] Rinella M.E. (2015). Nonalcoholic fatty liver disease: A systematic review. Jama.

[2] Draijer L., Benninga M., Koot B. (2019). Pediatric NAFLD: An overview and recent developments in diagnostics and treatment. Expert Rev. Gastroenterol. Hepatol..

[3] Sheka A.C., Adeyi O., Thompson J., Hameed B., Crawford P.A., Ikramuddin S. (2020). Nonalcoholic Steatohepatitis: A Review. Jama.

[4] Wong R.J., Aguilar M., Cheung R., Perumpail R.B., Harrison S.A., Younossi Z.M., Ahmed A. (2015). Nonalcoholic steatohepatitis is the second leading etiology of liver disease among adults awaiting liver transplantation in the United States. Gastroenterology.

[5] Cohen J.C., Horton J.D., Hobbs H.H. (2011). Human fatty liver disease: Old questions and new insights. Science (N. Y.).

[6] Schuppan D., Gorrell M.D., Klein T., Mark M., Afdhal N.H. (2010). The challenge of developing novel pharmacological therapies for non-alcoholic steatohepatitis. Liver Int. Off. J. Int. Assoc. Study Liver.

[7] Lazarus J.V., Mark H.E., Villota-Rivas M., Palayew A., Carrieri P., Colombo M., Ekstedt M., Esmat G., George J., Marchesini G. (2022). The global NAFLD policy review and preparedness index: Are countries ready to address this silent public health challenge?. J. Hepatol..

[8] Boccellino M., D’Angelo S. (2020). Anti-Obesity Effects of Polyphenol Intake: Current Status and Future Possibilities. Int. J. Mol. Sci..

[9] Sireesha Y., Kasetti R.B., Nabi S.A., Swapna S., Apparao C. (2011). Antihyperglycemic and hypolipidemic activities of Setaria italica seeds in STZ diabetic rats. Pathophysiology.

[10] Lu Y., Shan S., Li H., Shi J., Zhang X., Li Z. (2018). Reversal Effects of Bound Polyphenol from Foxtail Millet Bran on Multidrug Resistance in Human HCT-8/Fu Colorectal Cancer Cell. J. Agric. Food Chem..

[11] Ou Q., Zhang S., Fu C., Yu L., Xin P., Gu Z., Cao Z., Wu J., Wang Y. (2021). More natural more better: Triple natural anti-oxidant puerarin/ferulic acid/polydopamine incorporated hydrogel for wound healing. J. Nanobiotechnol..

[12] Liu Y.M., Shen J.D., Xu L.P., Li H.B., Li Y.C., Yi L.T. (2017). Ferulic acid inhibits neuro-inflammation in mice exposed to chronic unpredictable mild stress. Int. Immunopharmacol..

[13] Mu M., Zuo S., Wu R.M., Deng K.S., Lu S., Zhu J.J., Zou G.L., Yang J., Cheng M.L., Zhao X.K. (2018). Ferulic acid attenuates liver fibrosis and hepatic stellate cell activation via inhibition of TGF-β/Smad signaling pathway. Drug Des. Dev. Ther..

[14] Pei K., Ou J., Huang J., Ou S. (2016). p-Coumaric acid and its conjugates: Dietary sources, pharmacokinetic properties and biological activities. J. Sci. Food Agric..

[15] de Anda-Jáuregui G., McGregor B.A., Guo K., Hur J. (2019). A Network Pharmacology Approach for the Identification of Common Mechanisms of Drug-Induced Peripheral Neuropathy. CPT Pharmacomet. Syst. Pharmacol..

[16] Jacunski A., Tatonetti N.P. (2013). Connecting the dots: Applications of network medicine in pharmacology and disease. Clin. Pharmacol. Ther..

[17] Petroni M.L., Brodosi L., Barbanti F.A., di Domizio S., Petta S., Marchesini G. (2020). Lifestyle Changes for the Treatment of Nonalcoholic Fatty Liver Disease—A 2015-19 Update. Curr. Pharm. Des..

[18] Diehl A.M., Day C. (2017). Cause, Pathogenesis, and Treatment of Nonalcoholic Steatohepatitis. New Engl. J. Med..

[19] Wang B.H., Ou-Yang J.P. (2005). Pharmacological actions of sodium ferulate in cardiovascular system. Cardiovasc. Drug Rev..

[20] Liu F., Shan S., Li H., Shi J., Hao R., Yang R., Li Z. (2021). Millet shell polyphenols prevent atherosclerosis by protecting the gut barrier and remodeling the gut microbiota in ApoE(-/-) mice. Food Funct..

[21] Ed Nignpense B., Francis N., Blanchard C., Santhakumar A.B. (2021). Bioaccessibility and Bioactivity of Cereal Polyphenols: A Review. Foods.

[22] Awika J.M., Rose D.J., Simsek S. (2018). Complementary effects of cereal and pulse polyphenols and dietary fiber on chronic inflammation and gut health. Food Funct..

[23] Jahn D., Kircher S., Hermanns H.M., Geier A. (2019). Animal models of NAFLD from a hepatologist’s point of view. Biochim. Biophys. Acta. Mol. Basis Dis..

[24] Recena Aydos L., Aparecida do Amaral L., Serafim de Souza R., Jacobowski A.C., Freitas Dos Santos E., Rodrigues Macedo M.L. (2019). Nonalcoholic Fatty Liver Disease Induced by High-Fat Diet in C57bl/6 Models. Nutrients.

[25] Nanji A.A. (2004). Animal models of nonalcoholic fatty liver disease and steatohepatitis. Clin. Liver Dis..

[26] Oh Y.M., Kwon Y.E., Kim J.M., Bae S.J., Lee B.K., Yoo S.J., Chung C.H., Deshaies R.J., Seol J.H. (2009). Chfr is linked to tumour metastasis through the downregulation of HDAC1. Nat. Cell Biol..

[27] Wei W., Liu X., Chen J., Gao S., Lu L., Zhang H., Ding G., Wang Z., Chen Z., Shi T. (2017). Class I histone deacetylases are major histone decrotonylases: Evidence for critical and broad function of histone crotonylation in transcription. Cell Res..

[28] Kawano Y., Cohen D.E. (2013). Mechanisms of hepatic triglyceride accumulation in non-alcoholic fatty liver disease. J. Gastroenterol..

[29] Guo Y., Zhang X., Zhao Z., Lu H., Ke B., Ye X., Wu B., Ye J. (2020). NF-κB/HDAC1/SREBP1c pathway mediates the inflammation signal in progression of hepatic steatosis. Acta Pharm. Sin. B.

[30] Lai P.H., Wang W.L., Ko C.Y., Lee Y.C., Yang W.M., Shen T.W., Chang W.C., Wang J.M. (2008). HDAC1/HDAC3 modulates PPARG2 transcription through the sumoylated CEBPD in hepatic lipogenesis. Biochim. Biophys. Acta.

[31] Hao J.W., Wang J., Guo H., Zhao Y.Y., Sun H.H., Li Y.F., Lai X.Y., Zhao N., Wang X., Xie C. (2020). CD36 facilitates fatty acid uptake by dynamic palmitoylation-regulated endocytosis. Nat. Commun..

[32] Schwenk R.W., Holloway G.P., Luiken J.J., Bonen A., Glatz J.F. (2010). Fatty acid transport across the cell membrane: Regulation by fatty acid transporters. Prostaglandins Leukot. Essent. Fat. Acids.

[33] Yang C., Wan M., Xu D., Pan D., Xia H., Yang L., Sun G. (2021). Flaxseed Powder Attenuates Non-Alcoholic Steatohepatitis via Modulation of Gut Microbiota and Bile Acid Metabolism through Gut-Liver Axis. Int. J. Mol. Sci..

[34] Xie Z., Gao G., Wang H., Li E., Yuan Y., Xu J., Zhang Z., Wang P., Fu Y., Zeng H. (2020). Dehydroabietic acid alleviates high fat diet-induced insulin resistance and hepatic steatosis through dual activation of PPAR-γ and PPAR-α. Biomed. Pharmacother..

[35] Trott O., Olson A.J. (2010). AutoDock Vina: Improving the speed and accuracy of docking with a new scoring function, efficient optimization, and multithreading. J. Comput. Chem..

