# Supplementary material for: Ferulic Acid and P-Coumaric Acid Synergistically Attenuate Non-Alcoholic Fatty Liver Disease through HDAC1/PPARG-Mediated Free Fatty Acid Uptake
Source: Int J Mol Sci. 2022 Dec 4;23(23):15297. doi: 10.3390/ijms232315297 (PMC9736187; doi:10.3390/ijms232315297)

International Journal of *Molecular Sciences*

MDPI

Supporting Information

Supplement Figure 1 (A-F) The correlation analysis between HDAC1 and PPARA(A), SREBF1 (B), SREBF2 (C), NR1H3 (D), CEBPA(E) or FOXA2 (F).

Supporting Information

Supplement Figure 1

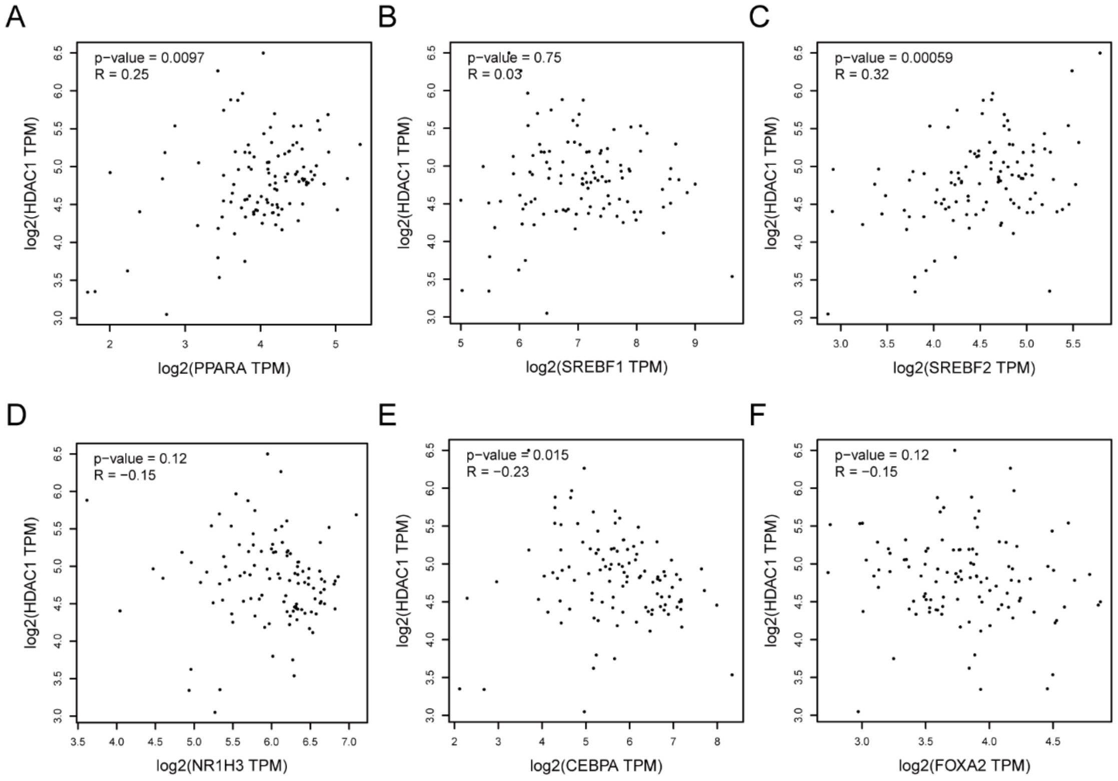

Supplement: Supplementary file 1 [file ijms-23-15297-s001.zip › ijms-2016223-Supplementary.pdf]
